# Rapid development of an integrated remote programming platform for neuromodulation systems through the biodesign process

**DOI:** 10.1038/s41598-022-06098-7

**Published:** 2022-02-10

**Authors:** Peter Silburn, Scott DeBates, Tucker Tomlinson, Jeremy Schwark, Gregory Creek, Hiren Patel, Asish Punnoose, Binith Cheeran, Erika Ross, Douglas Lautner, Yagna J. Pathak

**Affiliations:** 1 Queensland Brain Institute, The University of Queensland, QBI Building, 79, St Lucia, QLD 4072 Australia; 2 Abbott Neuromodulation, 6901 Preston Road, Plano, TX 75024 USA

**Keywords:** Biotechnology, Health care, Neurology

## Abstract

Treating chronic symptoms for pain and movement disorders with neuromodulation therapies involves fine-tuning of programming parameters over several visits to achieve and maintain symptom relief. This, together with challenges in access to trained specialists, has led to a growing need for an integrated wireless remote care platform for neuromodulation devices. In March of 2021, we launched the first neuromodulation device with an integrated remote programming platform. Here, we summarize the biodesign steps taken to identify the unmet patient need, invent, implement, and test the new technology, and finally gain market approval for the remote care platform. Specifically, we illustrate how agile development aligned with the evolving regulatory requirements can enable patient-centric digital health technology in neuromodulation, such as the remote care platform. The three steps of the biodesign process applied for remote care platform development are: (1) Identify, (2) Invent, and (3) Implement. First, we identified the unmet patient needs through market research and voice-of-customer (VOC) process. Next, during the concept generation phase of the invention step, we integrated the results from the VOC into defining requirements for prototype development. Subsequently, in the concept screening phase, ten subjects with PD participated in a clinical pilot study aimed at characterizing the safety of the remote care prototype. Lastly, during the implementation step, lessons learned from the pilot experience were integrated into final product development as new features. Following final product development, we completed usability testing to validate the full remote care system and collected preliminary data from the limited market release experience. The VOC data, during prototype development, helped us identify thresholds for video quality and needs priorities for clinicians and patients. During the pilot study, one subject reported anticipated remote–care-related adverse events that were resolved without sequelae. For usability analysis following final product development, the failure rates for task completion for both user groups were about 1%. Lastly, during the initial 4 weeks of the limited market release experience, a total of 858 remote care sessions were conducted with a 93% success rate. Overall, we developed a remote care platform by adopting a user-centric approach. Although the system intended to address pre-COVID19 challenges associated with disease management, the unforeseen overlap of the study with the pandemic elevated the importance of such a system and an innovative development process enabled us to advance a patient-centric platform to gain regulatory approval and successfully launch the remote care platform to market.

## Introduction

Neuromodulation therapies, such as deep brain stimulation (DBS), spinal cord stimulation (SCS), and dorsal root ganglion (DRG) stimulation, involve electrical stimulation of a neural target within a circuit to modulate neural activity^1–4^. DBS is currently approved to treat movement disorders, including essential tremor, and Parkinson’s disease (PD)^5,6^. For the treatment of pain, SCS is approved for chronic, intractable pain of the trunk and/or limbs, and DRG stimulation is approved for chronic, intractable pain of the lower limbs due to complex regional pain syndrome (CRPS)^7–9^. Although, technologies within neuromodulation (ex: lead geometry, battery performance, novel waveforms, and imaging techniques) are evolving rapidly^10^, treating chronic symptoms for pain and movement disorders still involves iterative fine-tuning of programming parameters. Stimulation programming depends on disease state and symptom profile and programming is an iterative process due to patient and response heterogeneity^11–13^. Additionally, trained specialists and care centers are typically located in dense cities, posing challenges for patients navigating urban transport systems or residing in distant areas. The need for iterative personalized programming evaluation coupled with travel challenges contribute to care access burden for patients and caregivers^13–16^.

Digital health, a rapidly expanding field centered on improving individuals’ health by combining technology with healthcare, including mobile health, wearable devices, and telehealth, can be employed to address disparities in access to care, cost, and travel burden associated with disease management^11,17,18^. It can be used to mitigate new challenges that have emerged due to the global COVID-19 pandemic. DBS, SCS, and DRG stimulation therapies can be optimized through digital health integration by leveraging remote real-time clinical assessment and dosing changes to affect clinical signs and symptoms. This integration can also foster patient personalization through continuous and objective measurement of parameters and facilitate data-driven clinical decision-making^10,19–23^.

### Significance

The healthcare burden for care access and disease management is constantly growing. Simultaneously, the acceptance of telemedicine solutions among patients has increased as demonstrated by recent market assessment data. The results of a recent survey (Abbott Survey; Table 1) indicate the need for tools that enable nuanced titration of DBS, SCS, and DRG therapy without increasing the burden on patients or clinicians. They also illustrate patient readiness to adopt a platform that can remotely connect them to experts and their existing doctors.

**Table 1:** Market Assessment of patient readiness to adopt telehealth technologies.

Adoptability of telehealth	SCS patients (%)	DBS patients (%)
Would reach out to doctor more with remote care feature when there was a problem with therapy	96	98
More hesitant to visit a doctor’s office since the global COVID19 pandemic	85	70
Use of telemedicine has increased since the global COVID19 pandemic	88	98

**Figure 1:**
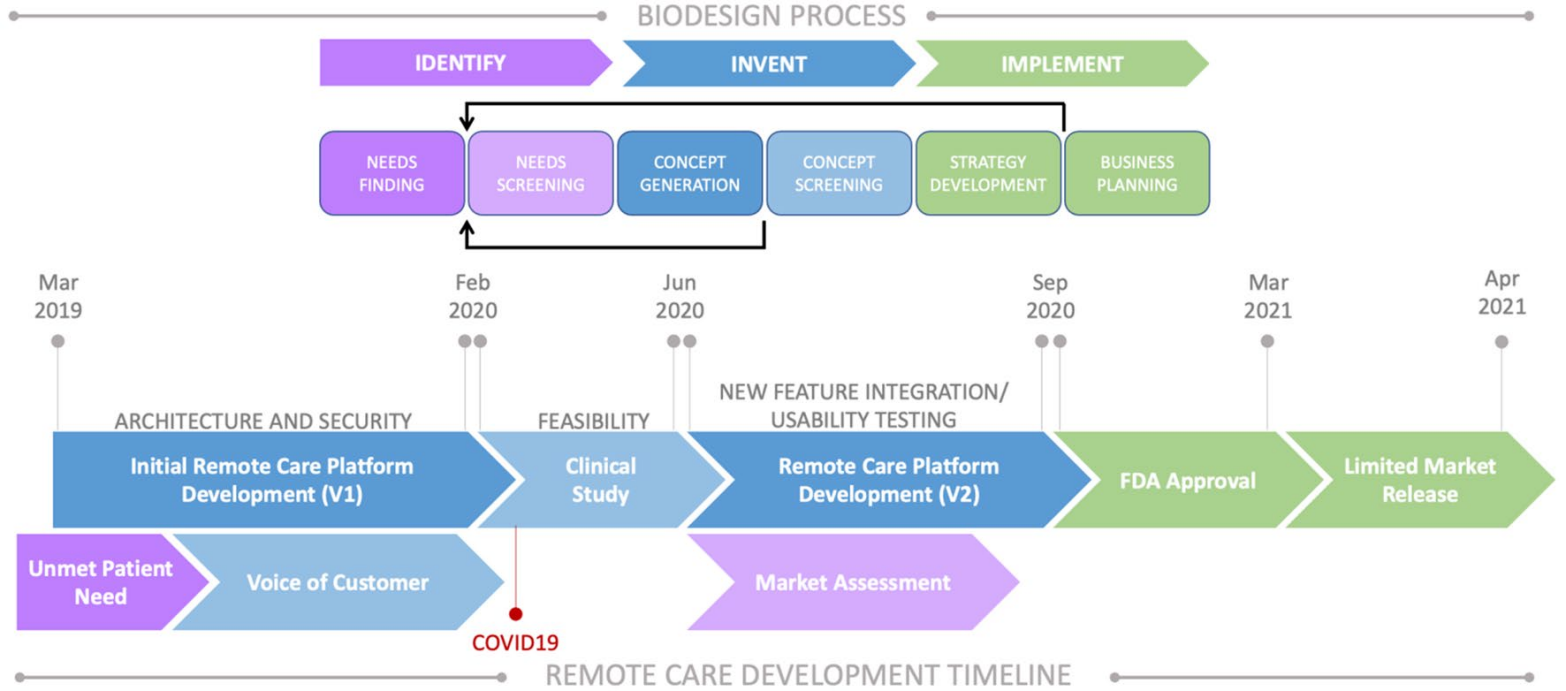
Development timeline of the Remote Care platform. The iterative development process of the remote care platform aligned with the biodesign process (adapted from Yock et al. 2015^24^). (1) We identified the unmet patient need related to care access burden; (2) We designed an initial prototype version of the platform in parallel with collecting voice-of-customer data and tested its feasibility in a real-world setting through a pilot study; (3) We integrated lessons learned from the study into a second iteration of development focused on new feature integration and usability analysis; (4) Lastly, we gained FDA approval for the remote care platform and collected preliminary data for the limited market release experience.

We began developing an integrated remote care platform prior to the COVID-19 pandemic to address the need for care access. However, we recognized the unmet need for this technology to be imminently useful in alleviating the added burden of disease management that emerged because of the global COVID-19 pandemic and expedited our investment resulting in regulatory approval and subsequent product launch in March 2021. Here, we present the biodesign-aligned path (Identify→Invent→Implement) we took to identify an unmet patient need, pilot and test a new technology, iterate the technology and validate with users, and finally, gain market approval^24^ (Fig. 1). The goal of the pilot study was to evaluate the prototype of the remote care platform (Phase 1) to characterize safety and determine its feasibility to enable expert programmers to remotely assess symptoms and adjust therapy settings in PD patients implanted with a DBS system. We integrated lessons learned from the study into subsequent development of the final product (Phase 2) and validated with SCS and DBS users through usability testing.

## Methods

In this section, we first outline the design process by reviewing key activities implemented. We detail the voice-of-customer (VOC) process in the identification step, expand on the concept generation and screening phase with protype development and the pilot experience in the invention step, and highlight new features integrated during final development that were evaluated with usability testing in the implementation step. We further present preliminary data from the limited market release experience. Lastly, we summarize the remote care platform and its notable features. All research activities we describe below, were performed in accordance with relevant guidelines and regulations.

### Identify

- *Voice-of-customer (VOC) and initial formative evaluation:* User feedback is essential to designing a relevant product. In an initial kick-off meeting with 9 clinician experts in neuromodulation, we worked through process maps and task-based scenarios to effectively identify user needs and pain-points. The goal of the initial formative evaluation during kick-off was to assess usability of the video-conferencing feature of the remote care application using qualitative feedback at variable levels of network quality. We tested prototype versions of the Clinician Programmer (CP) and Patient Controller (PC) applications for this activity with clinicians. Three pairs of CP-PC devices were programmed with varying levels of network throttling to determine the minimum acceptable video quality. Log files from the video-conferencing feature and usability survey data along with like/dislike tags were collected.
  For subsequent VOC activities, we conducted one-on-one sessions using tools from the structured quality functional deployment (QFD) approach to determine clinician and patient needs prioritization^25^. Twelve clinicians and 17 patients participated in this activity. Process maps included questions about (1) Patient scheduling, (2) Visit initiation/ system and device check, (3) Evaluation of therapy and patient outcomes with existing program, (4) Therapy adjustments and patient response, and (5) Documentation/follow-up visit plan. Individual and group needs statements were ranked by each clinician, normalized into scores, and averaged across the responders to determine overall hierarchy (Table 2). Clinician prioritization of each individual need was computed by multiplying the normalized within group ranking value with the ranking value the clinician assigned to the group as a whole, with the total of all needs equaling 100.

**Table 2:** Clinician needs grouping and ranked prioritization.

Group	Individual needs	Ranking (%)
Data and efficiency (36.4%)	Efficiently find effective settings	7.7
	Accurately manage patient data	3.8
	Efficiently manage patient data	2.3
	Maximize clinical value from patient interactions	4.6
	Efficiently manage clinic workflow	1
Performance and patient interaction (34.7%)	Efficiently address delayed onset symptoms	11.9
	Information to assess and minimize symptoms	8.0
	Information to assess and minimize side-effects	4.6
Financial (18.9%)	Reimbursement	18.3
Social (10.0%)	Social connection to patient	4.9
	Present a professional image	3.1

### Invent

**Figure 2:**
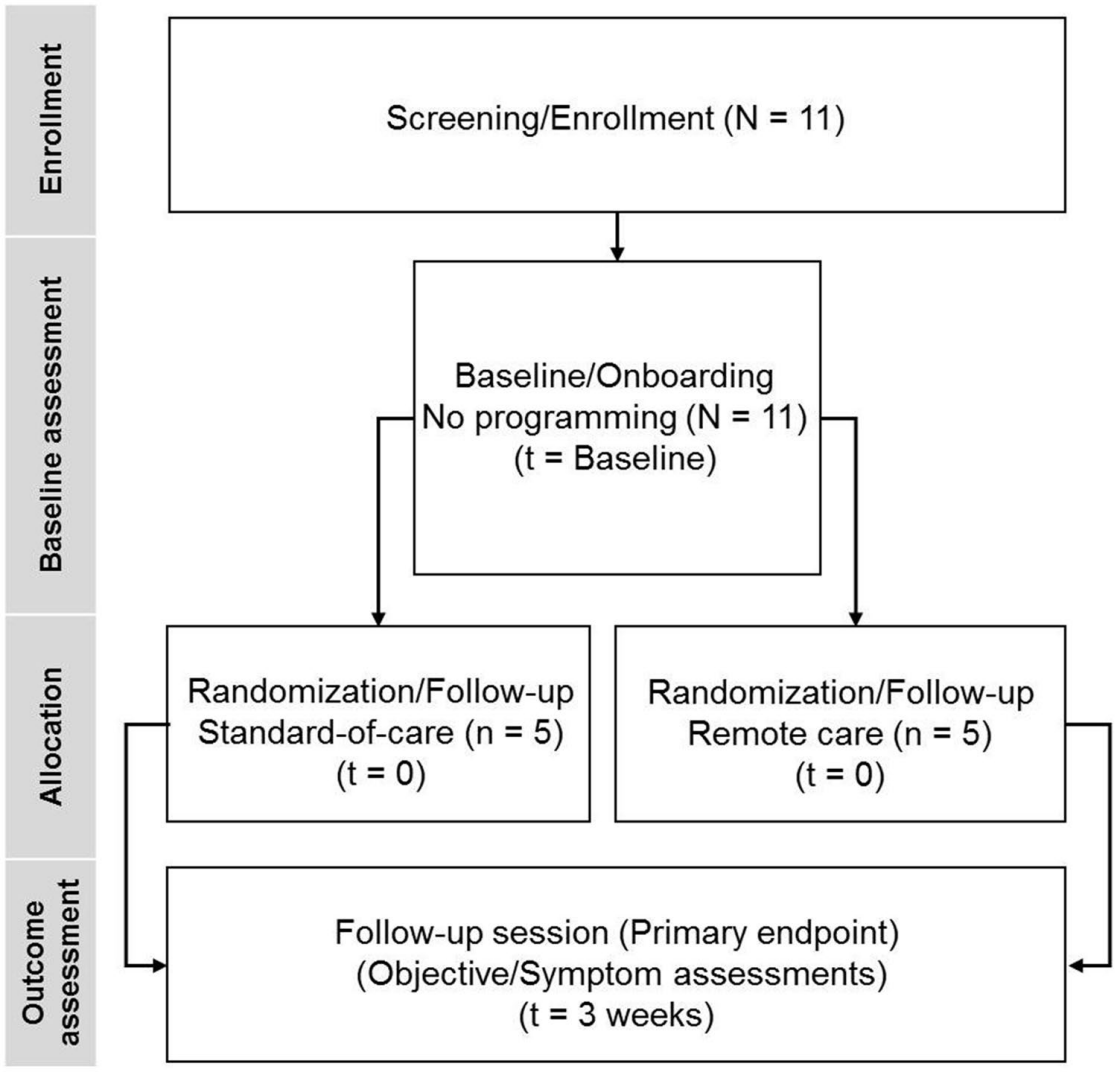
Flow diagram of the study design.
  *Screening/Enrollment:* Eleven subjects, who met the inclusion/exclusion criteria, were enrolled in the study. Only ten completed all clinical visits and one subject was withdrawn as they had not completed their randomization visit prior to COVID19 restrictions.
  *Baseline/Onboarding:* Following screening and enrollment, demographic details and other data pertaining to outcome measures were collected during the baseline assessment. Subjects were on-boarded through a hands-on training of the remote care platform that included highlights of the new features and a review of the updated user interface. At the end of the baseline visit, subjects received a remote care specific patient controller (PC), which was configured and paired with the clinician programmer (CP).
  *Randomization/Follow-up:* Enrolled subjects were required to be in the clinic for programming during the randomization/follow-up visit that was scheduled within two weeks of the baseline visit. The primary purpose of this visit was to assess the remote care platform and was not necessarily triggered by a programming need. The expert programmers assumed the role of being the in-person or remote clinician. Subjects were independently evaluated and programmed first by the in-person clinician and then by the remote clinician who was located in a different room or off-site. However, to ensure safety, the in-person clinician remained with the subjects during evaluation by the remote clinician in case any adverse events (AEs) occurred. Subjects were randomized in a 1:1 ratio to either the standard-of-care (SOC) group (n = 5) or the remote care (RC) group (n = 5), and only one set of parameters (either SOC or RC) was activated on the subject’s implantable pulse generator (IPG). A card containing the randomization code was provided to the site staff in a sealed envelope to determine the subject’s treatment group assignment. Both, the in-person and the remote clinician, were unblinded to the random assignment. A third assessor evaluated the subjects about 20 min after the programming session to determine the acute effects of the activated program. The third assessor and the subjects were blinded to whether the program was changed in-person or via the remote care platform. Additionally, site personnel were trained to ensure blinding was maintained. Programming information collected at this visit included session records indicating prior programming session details and session records after programming sessions were saved by the in-person and remote clinicians.
  *Primary endpoint:* The primary endpoint of the study was to evaluate the safety of the remote care platform through the characterization of programming-related AEs reported by the subjects within three weeks of the randomization visit. AEs were characterized as remote–care-related if they resulted because of the remote care feature. Anticipated AEs were defined as events that were expected and included in the protocol and consent form. All device-related AEs were recorded during the 3-week follow-up visit; however, only serious AEs were quantified towards the primary endpoint results. Programming was not required at this visit. If an in-clinic visit was not possible, AEs were captured remotely or over a phone call.
  *Statistical Analysis:* Descriptive statistics were used to characterize the AE profile of the remote care feature. There was no pre-specified hypothesis testing planned for this study.

- *Concept Generation (Prototype Development):* Processes to ensure quality were followed during the development process. Following planning activities, user needs were translated into customer, system, and product requirements. Designs aligned with established requirements were reviewed periodically during technical meetings. Additionally, we conducted risk management, hazard analysis and cybersecurity assessment prior to verification and validation to ensure that the remote care platform prototype met the required quality standards.
- *Concept Screening (Pilot Experience):* After prototype development, we conducted a pilot study in Australia to characterize safety, determine feasibility and collect real-world data to inform future improvements on the remote care platform. The study, focused on the DBS experience, was approved by the UnitingCare Health Human Research Ethics Committee. All subjects provided written informed consent which discussed potential risks and benefits of the device, study procedures, and compliance with review and follow up, prior to enrollment into the study (anzctr.org.au: ACTRN12619001660178).
  *Participants:* Subjects, aged between 21 and 75 years, who were diagnosed with PD and implanted with an Abbott Infinity™ DBS system (in STN) for at least 90 days were enrolled in this study. Additional inclusion criteria included: (1) willingness to maintain a constant dose of anti-PD medication indicated as best medical management for at least one month prior to study enrollment, (2) completion of at least one programming session and (3) availability for appropriate follow-up visits during the study period. Subjects were excluded if (1) they underwent DBS surgery within 90 days prior to screening, (2) had comorbid conditions or other medical, social, or psychological conditions that, in the investigator’s opinion, could limit the subject’s ability to participate in the clinical investigation or comply with follow-up requirements.
  *Study design:* Study design is depicted in Fig. 2 and summarized below.

### Implement

#### Final product development

- *Integration of new features:* Lessons learned from the clinical experience in the pilot study and the simultaneous environment of the pandemic inspired integration of additional features to the final product during Phase 2 of development. Notifications and support for user registration were added to enhance patient-clinician interaction during a remote therapy session. User portals enabling remote registration were put in place to further minimize travel burden. Using known concepts from cardiac stimulation devices, a network monitor to track if the patient or clinician has lost network communication or connectivity to the other device during the session was integrated into the remote care architecture. Also, we added a Protected Recovery Program (PRP) that enables the system to revert to a fail-safe program in the case of a network interruption during patient IPG programming^26^. Details about these features are outlined in sub-Sect. 4: Remote Care Platform and Notable Features.
- *Usability Testing:* Usability testing for the remote care platform was conducted remotely due to COVID19 restrictions, but in accordance with FDA guidance for Human Factors Validation Testing^27^.
  Clinicians participated in the usability testing, which comprised of 16 tasks across 4 use scenarios: (1) Set Up Clinician for Remote Care, (2) Mapping IPG to Clinician, (3) Remote Therapy Session, and (4) Troubleshooting. Patients completed 13 tasks across 4 use scenarios: (1) Set Up Patient for Remote Care, (2) Mapping IPG to Clinician, (3) Remote Therapy Session, and (4) Other. Subject matter experts on human factors process and the use of neurostimulation products in typical clinical use environments, facilitated remote test sessions with clinicians and patients via a web-based meeting platform (ex: WebEx). At least two researchers (one moderator and at least one observer) were present during the session with the respective participants. Simulated-use testing was used; the nature of user interactions with the Remote Care feature and device application (CP or PC) enabled a thorough evaluation under simulated, naturalistic conditions.
  The Remote Care CP application was installed on a CP device (iPad Mini™) before the device was sent to the clinician; the installed application was managed by AirWatch™, a mobile device management platform, to ensure access only to appropriate users, and to allow for application updates, if needed. In this set-up, the PC device was controlled by the moderator and paired with a mock IPG (Infinity™ DBS or Proclaim™ SCS), that was connected to therapy-specific leads, implementing a fully functional system for testing. The moderator presented the clinician with a task which was completed using the Remote Care CP application within view of the video so that the moderator and observer(s) could note the interaction.
  The Remote Care PC application was delivered to the patient’s PC device (iPod Touch™ or qualified iPhone™) remotely via AirWatch™ to ensure access only to appropriate users, and to allow for application updates, if needed. In this set-up, patients used the PC application’s “demonstration mode” to complete all tasks; the demonstration mode simulated connection to an IPG—patients were not allowed to connect the Remote Care PC application to their actual IPG accounting for safety. The moderator presented the patient with a task which was completed using the Remote Care PC application, within view of the video so that the moderator and observer(s) could note the interaction. Test sessions, for both clinicians and patients, lasted approximately one hour.
  The tasks, specific use scenarios, and associated acceptance criteria are summarized in Table 3 (clinicians) and Table 4 (patients). Within these scenarios, tasks were considered critical if they were associated with moderate or high severity. For example, an error that could result in unintended stimulation effects for the patient was considered critical.

**Table 3:** Task description and use scenarios for usability testing with clinicians.

Task	Acceptance criteria
**(1) Set up clinician for remote care**	
Set up remote care on the clinician programmer	Launch the Remote Care application
Clinician RTB registration	Access clinician registration screen
Clinician login to remote care	Enter provided credentials and login to Remote Care account
**(2) Mapping IPG to clinician**	
In-person mapping	Enable Remote Care feature on demonstration IPG
**(3) Remote therapy session**	
Establish remote connection to IPG	Successfully connect to Remote Care session with moderator
Manage protected recovery program	Identify current protected recovery program
	Set new protected recovery program. Set desired stimulation state for protected recovery program
Clinical evaluation of patient	Able to see, hear, and communicate with Moderator through Remote Care session
	Expand/minimize full screen video
System integrity check	Run system impedance check
Adjust stimulation	Hide/show patient video screen
	With the minimized video screen, users must: adjust amplitude, pulse width, and frequency; turn stimulation on and off; and change lead electrode configurations
	With the full video screen, user must adjust amplitude, pulse width, and frequency and turn stimulation on and off
View patient/generator information and logs	Identify generator battery health status
End a remote care session	End session and save programming changes using the “End Session” button
**(4) Other**	
Troubleshooting	Successfully answers knowledge questions regarding session disconnects

**Table 4:** Task description and use scenarios for usability testing with patients.

Task	Acceptance criteria
**(1) Set up patient for remote care**	
Set up remote care on the patient controller	Install Mobile Device Management (AirWatch)
Locate a bluetooth connection on your PC device and “forget” that connection	Can walk through removing a Bluetooth connection
Create a new Bluetooth connection between your generator and your PC device	Can walk through creating a Bluetooth bond
**(2) Mapping IPG to clinician**	
Remote mapping	Launches the correct application (Remote Care) Identify model number and serial number of demonstration IPG
**(3) Remote therapy session**	
Establish remote connection to IPG	Successfully connect to Remote Care session with moderator
Clinical evaluation of patient	Able to see, hear, and communicate with Moderator through Remote Care session
	Expand/minimize full screen video
End a remote care session	Tap on "End Session"
End session prompt	Participant indicates that programming changes would be lost, or therapy would revert back to a prior setting
If you accidentally tapped on the End Session button	Participant indicates that they would select “Cancel.”
Save the programming changes	Participant indicates that the clinician needs to end the session
**(4) Other**	
Session Ended Unexpectedly: What does the error message mean?	Participant indicates that there was an unexpected session disconnect
Session Ended Unexpectedly: What do you believe happened to your program settings in this case?	Participant indicates that therapy reverted or was restored; Participant indicates that program changes were not saved
In the case of an unexpected disconnect, what would you do?	Participant indicates they would reconnect to the clinician; Participant indicates they would get in contact with the clinician

#### Limited market release

On March 23rd, 2021, we released the remote care platform to a limited number of sites. Preliminary data measures about user analytics and session quality were collected from the backend server.

### Remote care platform and notable features

#### Interface

A remote interface for the Abbott DBS and SCS systems (Infinity™/Proclaim™, St. Jude Medical, USA) was developed by leveraging the existing multi-component technological platform. Clinicians can use the interface on their iPad CP to perform programming and analysis as a telehealth service. The iOS-based remote interface integrates with DBS or SCS systems through existing in-built Bluetooth® communication between the PC and the IPG. Mobile device camera and microphone, native to the user devices, are used from both end points to establish a live audio-video conference and enable clinicians and patients to interact effectively during a remote therapy session.

Clinicians and patients can interface with the platform via a single device at their respective ends; clinicians interact with the clinician programmer (CP, Apple iPad) and patients with their patient controller (PC, Apple iPod touch provided at the time of implant or patient’s personal iPod or iPhone). The CP and PC connect remotely over cellular or Wi-Fi connection, enabling the clinician to simultaneously adjust therapy on the IPG and digitally prescribe parameters through mobile applications installed on the CP. The focus of this design was to provide an efficient and secure therapy session by optimizing the remote therapy backend infrastructure while keeping the interface simple.

**Figure 3:**
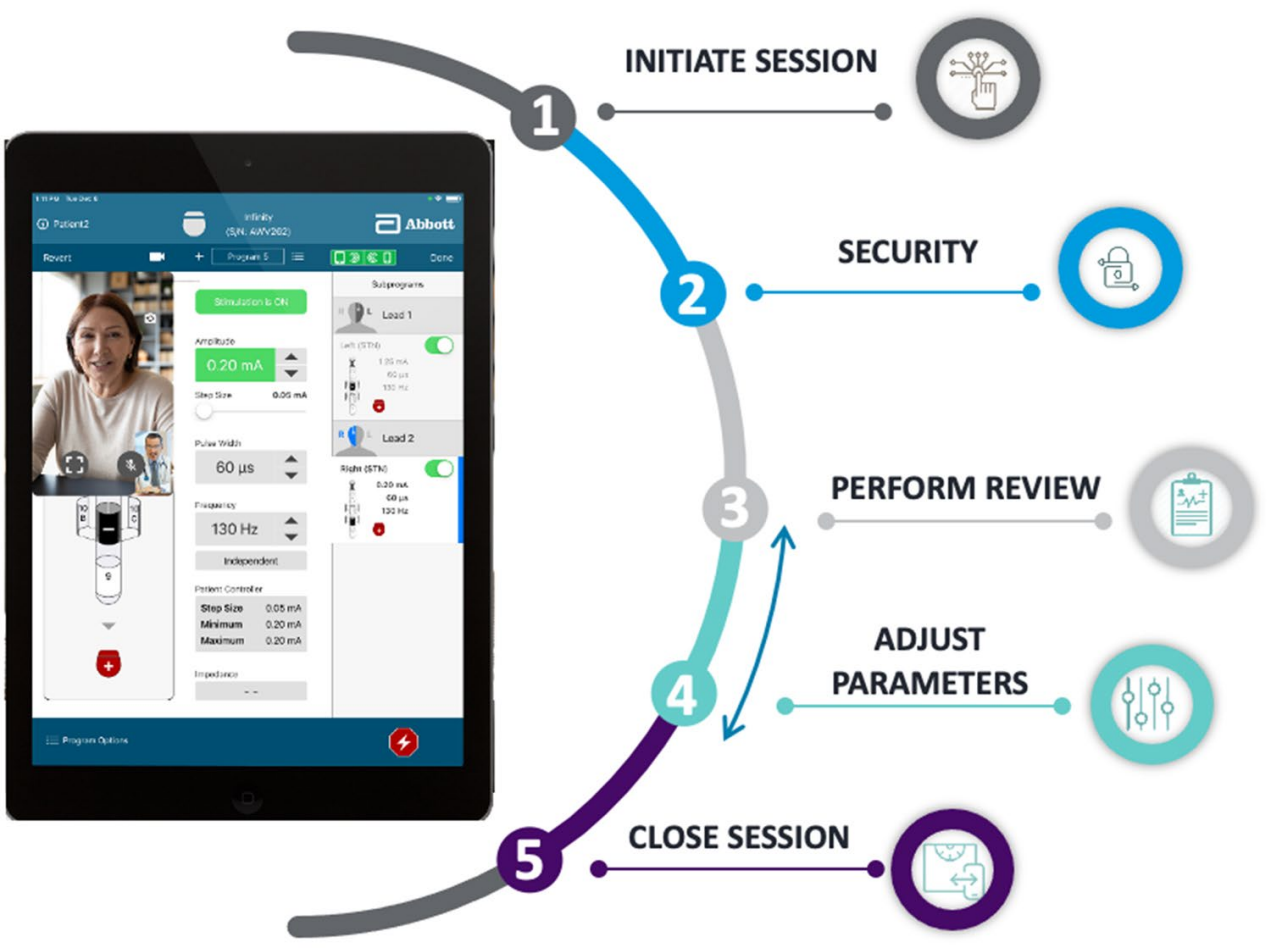
Clinical and communication workflow for a remote session. A typical remote care session using the remote care platform consists of the following steps: (1) session is initiated by the subject, (2) authentication of users and devices enabled via secure connection through the backend server, (3) review of symptoms and performance by the clinician, (4) iterative adjustment of programming parameters including amplitude, pulse width, frequency, and contact selection, (5) settings are saved, and session is closed by the clinician. *Individuals portrayed in the figure are not actual patients. Images are representative and taken from stock photos (https://www.shutterstock.com/image-photo/head-shot-mature-woman-looking-camera-1720702294).

A typical programming session can be categorized into 5 steps (Fig. 3): (1) Session initiation, (2) Security exchange, (3) Perform review, (4) Adjust parameters, and (5) Save and close session. While the steps remain consistent between the remote and in-person implementation, the remote care scenario is adapted to be compatible with the digital environment.

#### Privacy and cybersecurity

**Figure 4:**
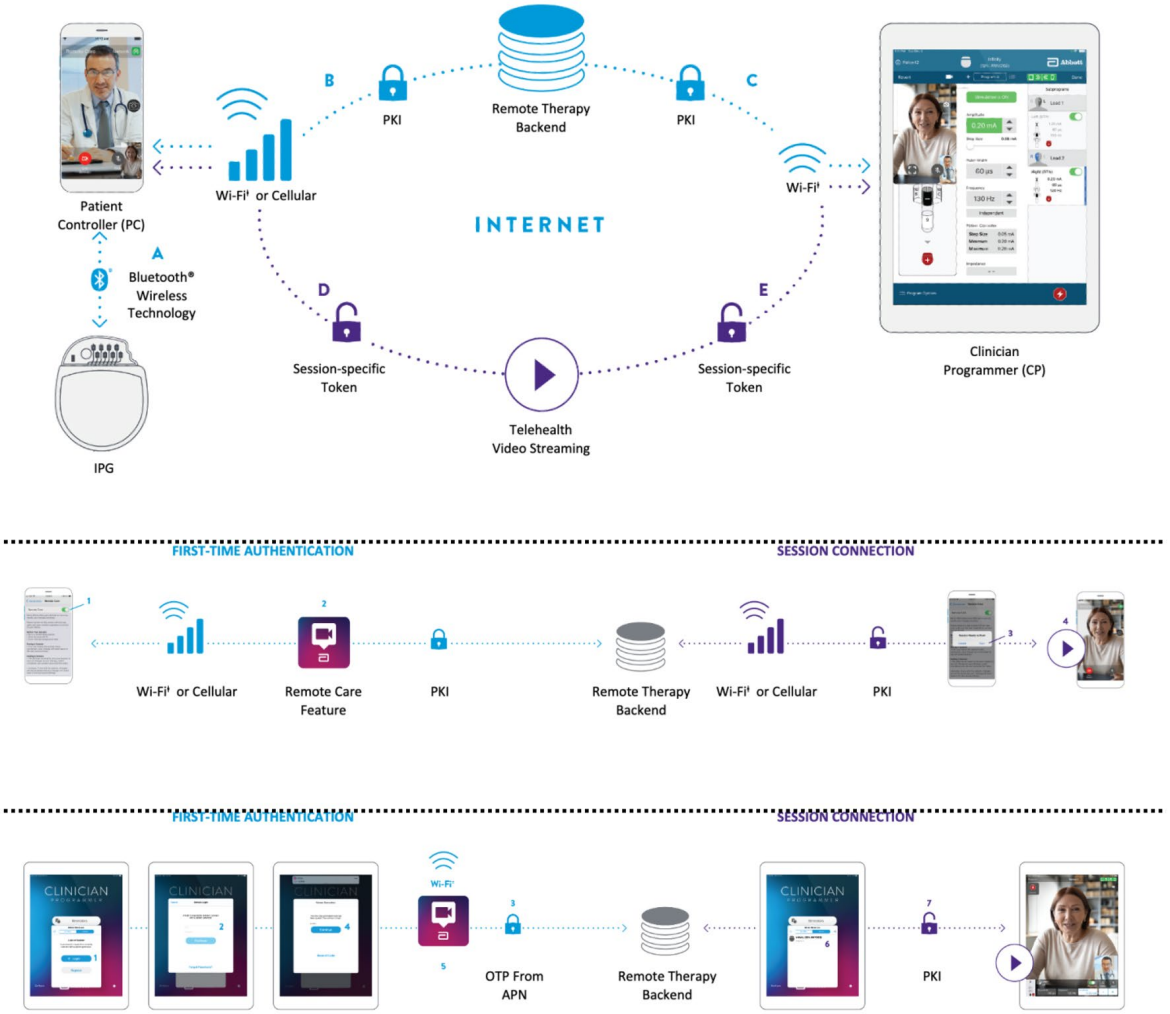
Remote care platform architecture and communication pathways. *Top:* There are two communication pathways integrated into the remote care platform: device programming pathway (blue) and video communication pathway (purple). (**A**) Two-way Bluetooth wireless technology-enabled communication between IPG and PC. (**B**) Two-way Wi-Fi‡ or cellular communication of data over the patient’s network between the PC and Remote Therapy Backend; data are protected through PKI. (**C**) Two-way Wi-Fi‡ communication of data over the clinician’s network between the CP and Remote Therapy Backend; data are protected through PKI; CP does not communicate directly with the patient’s IPG during a remote session. (**D**) Two-way Wi-Fi‡ or cellular communication of video streaming data over the patient’s network between the PC and video streaming service; session-specific token issued for each video session. (**E**) Two-way Wi-Fi‡ communication of video streaming data over the clinician’s network between the CP and video streaming service; session-specific token issued for each video session. *Middle****:*** Connection from the patient perspective is divided into first-time authentication (Blue; step 1: Remote Care Feature enablement and step 2: Remote Therapy Backend enablement) and session connection (Purple; step 3: PC app availability is advertised for a Virtual Clinic session and step 4: video data session is established). *Bottom:* Connection from the clinician perspective is divided into first-time authentication (Blue; steps 1–5) and session connection (Purple: steps 6–7): step (1) User registers the CP app for the Virtual Clinic; step (2) User creates username and password; step (3) Remote Therapy Backend verifies proof of possession of the Remote care app running on the CP; step (4) User receives one-time password (OTP) enters it into the CP app; step (5) CP app authentication occurs using PKI step; (6) CP app interrogates the Remote Therapy Backend for available IPGs registered to the clinician and sees the IPG; and (7) Remote Therapy Backend stages a session for video data to set up a video chat. CP, clinical programmer; IPG, implantable pulse generator; PC, patient controller.

The remote care platform was developed on a secure remote therapy backend (RTB) server that connected components of the existing DBS system to facilitate patient evaluation through this novel remote platform (Fig. 4). The platform employs two levels of connectivity: (1) Bluetooth® connectivity that enables the PC to connect with the patient’s IPG, and (2) network connectivity that connects the CP with the PC through Wi-Fi or cellular data.

The CP application is authenticated by the backend using multifactor authentication and requires the use of a 14-character password. The CP-PC communication is facilitated via the secure RTB server; it uses mutual end-to-end authentication based on Public Key Infrastructure (PKI) credentials provided at the time of initial registration between the two applications. Additionally, all communication between the CP and PC is protected via Advanced Encryption Standard (AES) 256 encryption. The remote care platform is architected to use the patient’s controller application (Remote Care PC application) as a proxy for receiving therapy commands sent by the clinician’s programming application (Remote Care CP application) over a network connection. Upon receipt of a validated command, the Remote Care PC application will send the program settings to the patient’s IPG over Bluetooth® connection. The PC application is authenticated using the patient’s IPG as a token. The Bluetooth® connection between the PC and IPG is maintained as in the conventional, in-person system. Before Bluetooth® technology communication channels can be established, devices must be paired or bonded. This handshake process allows two devices to identify, settle on how to communicate with, and remember each other. This bonding process needs to be initiated with a designated magnet applied to the IPG with the PC within 2 meters of the IPG. Only one PC is allowed to bond to an IPG. Further, all communication involving the CP, PC and IPG use a proprietary protocol and is secured using Advanced Encryption Standard AES 128 encryption. All data, including data at rest and in transit from the IPG, are encrypted with strict access controls applied. Additional details of the security protocols employed were reviewed and approved by FDA and other global regulatory bodies (ex: Health Canada, EU regulators, etc.). Information about Abbott’s privacy policy can also be found at: https://www.abbott.com/privacy-policy.html.

In summary, the backend infrastructure of the remote care platform is built with a security-first approach to safeguard remote care sessions and patient data; the security architecture of the system is designed to comply with internationally recognized security standards, and with evolving data privacy laws so it is configurable and deployable around the world. System and organizational control (SOC-2) certified tools (Microsoft Azure cloud) are leveraged for configuration. The platform uses a defense-in-depth approach configured with multiple layers of security, as described, to protect data at rest and in transit and to secure a chain of trust end-to-end between the edge devices as they communicate through the backend infrastructure.

#### Safety-first

Two specific features are integrated to account for the robustness of network connectivity to ensure safe implementation of the remote care session. (1) A color-coded network indicator, on the CP and PC application interfaces, enables the patient and clinician to monitor reliability of their session connection. Additionally, the CP application interface also displays the patient’s network state so the clinician can consider that information before making drastic adjustments. (2) Protected Recovery Program (PRP) refers to defining a fail-safe program for recovery operations. This PRP serves as the default that the patient’s device can revert to, if an unexpected network interruption occurs. By default, a copy of the active program at the beginning of a session is defined as the PRP. However, the clinician can assign a new and optionally customized program as the PRP, if preferred. During an unanticipated interruption (ex: network loss) or a patient-forced termination of the session, the patient device will automatically revert to the PRP (does not need to be triggered by patient or caregiver) to ensure that the patient is not left with a sub-optimal program.

## Results

### VOC and initial formative evaluation results

#### Initial formative evaluation

One of the goals of the initial formative evaluation was to determine the threshold for acceptable quality of video for clinicians to reliably assess the patient remotely. Video quality is related to network conditions, hence, bit rate parameters in the logs were used to correlate with like and dislike tags. Our results showed that the like tags were evenly distributed, however, the dislike tags were clustered for values below 1,000,000.

#### One-on-one sessions

During one–one–one sessions, clinicians were asked to first compare needs at the group level (Data & Efficiency, Financial, Performance and Patient Interaction, and Social), followed by comparisons within each group. Needs within each group are summarized in Table 2. In terms of group-level ranking, Efficiency and Data (36.4%) and Performance and Patient Interaction (34.7%), were at the top. For individual needs statements, reimbursement (18.3%), efficiently addressing delayed onset problems with therapy (11.9%), and information to address and assess symptoms (8.0%) ranked at the top.

During patient interviews, 58% of the patients reported having to travel more than 50 miles or over one hour for their programming visits. All of them reported owning a smartphone or a tablet, and 92% reported having access to Wi-Fi at home. About 81% of the patients reported experience with video-conferencing in the past and 86% reported that a remote care solution would be appealing for either some or all their visits.

### Pilot study

Eleven subjects with PD were enrolled in this study, however, only ten completed the randomization and primary endpoint visits (male = 7; female = 3). The last subject was withdrawn before their randomization visit due to COVID19 shutdown. For the remaining ten subjects, the mean duration of PD for all subjects was 11.8 years since the symptom onset (range 6–17 years) and 10.5 years since the first diagnosis. There were no significant differences between the SOC and RC groups in the mean duration of diagnosis (SOC: 9.8 [6.59–13.01]; RC: 11.2 [6.13–16.27]; *P* > 0.05) or mean duration since symptom onset (SOC: 12.2 [8.75–15.65]; RC: 11.4 [6.24–16.56]; *P* > 0.05). Primary symptoms for the subjects included difficulty walking (n = 5; SOC = 2, RC = 3), rigidity/stiffness (n = 2; SOC = 1; RC = 1), feeling tired (n = 1; SOC = 1), slow movements/bradykinesia (n = 1; SOC = 1), and slurred speech (n = 1; RC = 1). Average time elapsed since the DBS implant for all subjects was 433.3 days (range 96–716 days). For all subjects, the leads (left and right) and the IPG were implanted on the same day.

One subject reported 4 remote–care-related AEs (tremor, rigidity, cramps, and foot pain) during the randomization visit that were quantified towards the primary endpoint. These AEs were reported as worsening of existing condition. However, all AEs were resolved without sequelae. Specifically, the events described resulted from loss of connectivity while alternate settings were being evaluated. The session was force-closed during the transfer, which resulted in settings changed by the remote clinician during the interrupted session to be saved on the PC in the “off” state. Within minutes of the interrupted session, the subject’s symptoms appeared and immediate intervention by the in-person clinician reversed the AEs by turning DBS “on”. AEs reported within the study were as anticipated for the study population. The study protocol identified the scenario as a foreseeable risk: unintended stimulation effects due to communication loss between the remote clinician and the patient.

### Usability testing

Based on the Human Factors Validation testing, the Remote Care applications for the CP and the PC were demonstrated to be safe and effective for intended users, uses, and use environments.

Specifically, for the CP, 3 task failures were observed. The failures were associated with tasks in the troubleshooting category and would not have led to patient harm. The risk of patient harm was determined by reviewing the probability of an adverse health outcome defined based on past evidence.

Of the observations not associated with task failures, most were navigation errors—within the application, participants looked in different locations for the feature or UI element needed to complete the task. These navigation errors were corrected immediately and had minimal disruption in the task workflow. Navigation errors occurred when participants attempted to modify the program that would be applied to the IPG in the event of a dropped network connection. The inability to modify that program in the event of a dropped network connection would not have resulted in patient harm, as the active program is automatically protected at the beginning of the session. When asked, clinicians did not indicate that any elements of the Remote Care feature were concerning. All clinicians were able to end the remote care session appropriately. Though 2 of the 16 clinicians misinterpreted the alerts for disconnect prompts, all clinicians understood what happened to the patient’s program settings and responded appropriately when asked what they would do after an unexpected disconnect. The features and tasks in place resulted in appropriate behavior from clinicians and effectively managed potential harm associated with unexpected disconnects.

For the PC, design features in place were found to be acceptable and there were no failures of critical tasks. Patients were able to successfully complete all provided tasks, including setting up the Remote Care PC application on their device and conducting a remote care session. Three instances of task failure were observed, with two having the potential to result in patient harm if they were unable to adjust their therapy settings. The majority of observations, including two of the task failures, were observed during Use Scenario 1—Set Up Patient for Remote Care. The most likely outcome of any failures during set up is that the patient would not be able to utilize the Remote Care feature until they received assistance with the setup process.

### Limited market release

**Figure 5:**
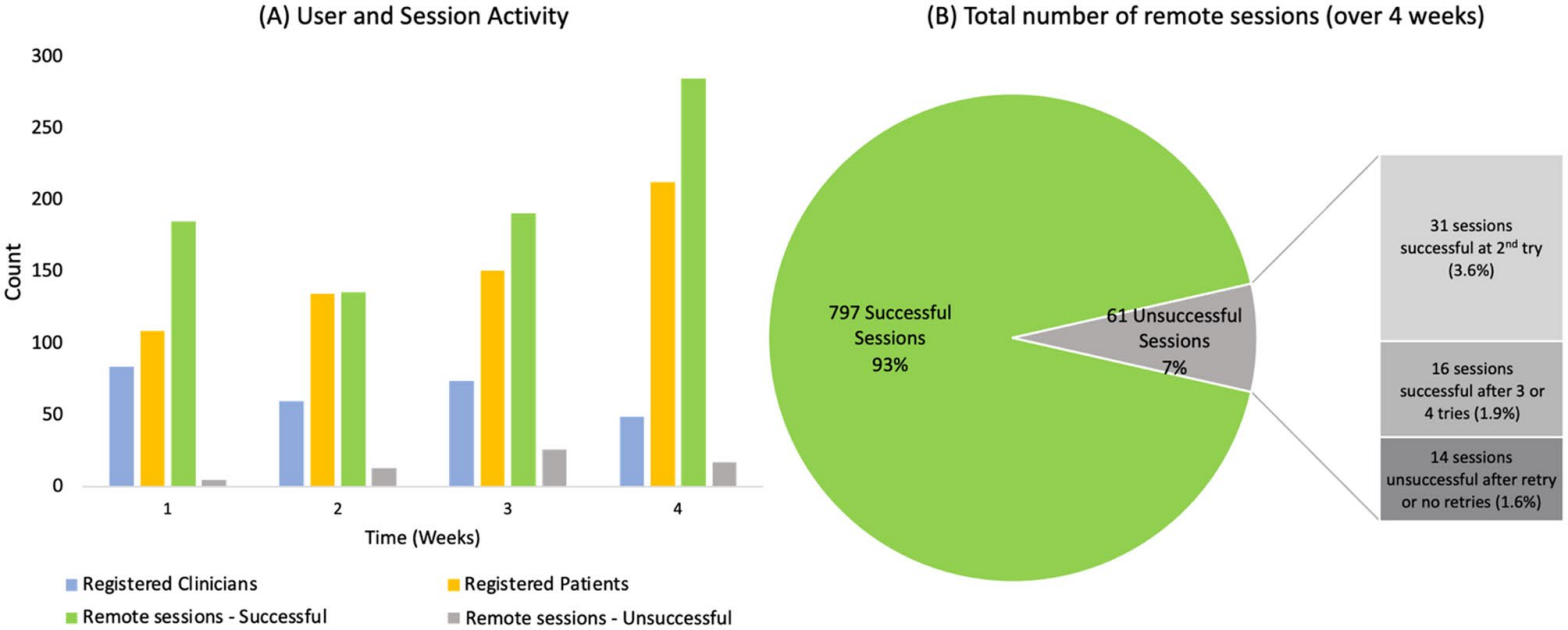
User analytics and session quality data from 4-week preliminary experience during the limited marker release of the remote care platform. (**A**) Representation of registered clinicians and patients in the user portal and the number of remote connections. (**B**) Number of successful and unsuccessful remote session connections with a breakdown of retry status.

We aggregated data about user analytics and session quality from the experience within the first 4 weeks after launch (Fig. 5). During this time, 267 clinicians and 608 patients registered for remote care using the user portal, and a total of 858 sessions were conducted. Of these sessions, 797 were successful connections (~ 93%) while 61 were unsuccessful connections (~ 7%). About half of these unsuccessful connections were successful on the second try (31 sessions (~ 3.6%)) and about a third were successful on the third or fourth try (16 sessions (~ 1.9%)). Hence, the total number of sessions that account for unsuccessful connections after retry or no attempted retries is 14 sessions (~ 1.6%). The data is summarized in Table 5 with a detailed breakdown of the unsuccessful connections in Table 6.

**Table 5:** Preliminary data from Limited Market Release of the remote care platform.

Week	Registered clinicians	Registered patients	Remote sessions successful	Remote sessions unsuccessful
1	84	109	185	5
2	60	135	136	13
3	74	151	191	26
4	49	213	285	17
Total	267	608	797	61

**Table 6:** Breakdown of the failed remote sessions.

Session status	Unsuccessful sessions	Number of devices
Successful after 2nd try	31	31
Successful after 3/4 tries	16	15
Unsuccessful or no retries	14	
Total	61	46

## Discussion

Here, we summarized the development of the remote care platform aligning with the 3-step iterative biodesign process. We collected VOC data during prototype development to identify user needs and aligned our development efforts accordingly. Subsequently, we tested the remote care platform in a clinical environment with a proof-of-concept study to establish feasibility and characterize safety in a real-world setting. We then integrated features based on lessons learned from the study into subsequent development which was validated along with the workflow with human factors usability testing. The development efforts resulted in first regulatory approval (in the United States) and subsequent product launch of the remote care platform in March 2021 (Fig. 1). This was followed by CE-mark and TGA approvals of the remote care platform leading to product launch in Europe and Australia in October 2021.

The VOC data, during prototype development, helped us identify thresholds for video quality and needs priorities for clinicians. The video quality stress test demonstrated that to be below the bit-rate threshold, artificial throttling, not usual in typical use cases, was required. Through patient interviews, we established that patients are familiar with base technology upon which the remote care platform was developed. Specifically, responses relating to experience with video-conferencing enabled the development team to design an interface that would be easy-to-adopt for patients. These findings served as the foundation for a user-centric design of the remote care platform.

In the pilot study, where patients and clinicians engaged with the remote platform in a real-world setting, ten subjects were able to successfully connect with their remote clinician using the remote care platform. The remote clinician could modulate a range of programming parameters including, amplitude, frequency, pulse width and contact selection (longitudinal, radial, and directional) in real time simultaneous with audio-visual assessment. The remote clinician also tested the feasibility of this system with successful connections from variable settings (a different room in the clinic, a remote urban and a rural setting). The primary objective of the study was to characterize the safety of remote–care-related AEs; therefore, a group-level comparison between the RC and SOC groups was not within the scope of this study. During the primary endpoint duration, only one subject reported remote–care-related AEs, specifically due to loss of connectivity between the CP and PC devices. The AEs experienced by this subject were anticipated, categorized as *worsening of the clinical condition*, and resolved without sequalae. Protected recovery program in the prototype version of the platform, employed in study, was defined as the last saved checkpoint on the IPG. For this patient, the checkpoint was saved with DBS off, resulting in the observed adverse event. This PRP feature was improved during final product development to enable the clinician to define a more optimal program. There were no unanticipated or serious AEs reported during the study.

During the final product development, novel features were integrated to mitigate the challenges identified through VOC analysis and real-world feasibility data from the pilot experience. The usability analysis also demonstrated that the workflow associated with the addition of the remote care feature can be seamlessly adopted by patients and clinicians. For both populations, the failure rate for tasks was about 1%, implying that with increased use and familiarity, this rate may drop further. Additionally, none of the failures occurred for tasks that could lead to patient harm.

The development culminated into a limited market release of the remote care platform. A preliminary assessment of the data collected within the first 4 weeks demonstrates the performance of the remote care system in an uncontrolled setting. Additionally, it underscores the value of a scalable backend. Though the users supported tripled the anticipated number in the first four weeks, the rate for unsuccessful remote session connections remained low. Lastly, the increased number of users further illustrates the growing need and overall willingness to adopt this digital health technology to mitigate current and previously existing challenges with neuromodulation care.

We developed the remote care platform, currently available on iOS, to address the growing care access burden for patients, clinicians, and caregivers. The platform was uniquely architected to leverage existing mobile platforms without the need for any extra hardware components, enabling a shallow learning curve for patients and clinicians. External sensors, such as built-in mobile cameras, enabled high-resolution audio–video sessions and facilitated real-time evaluation of symptoms. Our development efforts were iterative; we identified an unmet patient need with respect to care access burden, designed the initial technology for use in the pilot study and subsequently, integrated features informed by lessons learned in the study to advance towards a patient-centric remote care platform that was further evaluated with a preliminary limited market release experience. In the study, issues related to connectivity were most common among the technical concerns related to the platform. In response, the PRP feature was refined to address unanticipated network interruptions, and a color-coded network monitor was added to the UI to mitigate the need for robustness of system connectivity. Additionally, nuances of system usability were refined for a more robust and comfortable remote care experience. Usability testing, pilot testing, and real-world adoption demonstrate that the system provides a mechanism for patients to access diagnostic or treatment services for their neuromodulation system when they do not have access to in-person services. Further, use of the system may be expected to provide rapid treatment in cases where in-person treatment is delayed (ex: patients needing to travel long distances to see their care provider).

Technological progress in the field of neuromodulation has thus far depended on hardware-based improvements such as implantable device miniaturization and improved geometry of electrodes. However, currently, there is a need for digital solutions that can extend care beyond the clinic to address growing care-access burden and to evaluate patient concerns in real-world scenarios. Previous systems that have attempted to remotely connect patients and clinicians are cumbersome and often contain multiple components. Most systems only provide a video platform, requiring the patient to adjust their own programming settings within previously programmed parameter ranges. Other platforms, such as the PINS and SceneRay systems recently investigated in China, require multiple hardware components to integrate the video interaction with therapy adjustments^28,29^. In contrast, our remote care system leverages existing mobile technology to initiate an audio/video interaction simultaneously to activating a data channel for sending therapy commands. Though two channels are activated to ensure safety of data while in rest or transit, the users only need to interact with a single interface, minimizing overall burden of use. Moreover, our platform can be used with patient’s personal mobile devices (ex: personal iPhone) further minimizing the user burden of multiple device management. Currently, there are no other remote care systems that are completely wireless, and enable simultaneous modulation and monitoring as demonstrated in the platform presented here^28,29^. Novel technical features integrated in the remote care system described, and the overall user experience, are focused on empowering the patient during the remote session: (1) Patient-initiated process ensures that the patient is always in control of their therapy; (2) Addition of remote care features on existing interface allows for seamless adoption of the new platform; (3) Safety-first foundation of the system is architected with multiple layers of security to adequately protect patient data and session communication; (4) Color-coded indication about session connectivity is presented with the network monitor to communicate the quality of the connection; (5) Ability for the clinician to comprehensively prescribe programming changes remotely to account for fluctuating symptoms and side-effects; and (6) A fail-safe mechanism with the PRP feature that can be customized by the clinician to ensure that patient is on optimal settings regardless of unanticipated communication loss during the session.

Certain limitations were associated with the pilot study; it only evaluated the feasibility of the remote care platform and characterized the remote–care-related safety. Future studies, that are appropriately powered will characterize the clinical, psychosocial, and economic benefits over time with the use of the remote care platform. Additionally, COVID-19-related restrictions led to several disruptions during the data collection phase. Endpoint measures at the 3-week visit were captured over the phone for many subjects due to pandemic-related travel and clinic attendance restrictions. However, the pandemic has emphasized the importance of such remote care platforms and the need to address the imminent challenge of safely accessing healthcare options. The crisis has impacted the management of several chronic conditions, including PD and chronic pain, and has magnified the existing limitations several folds. Visiting care providers for a routine check or iterative adjustments to programming settings is measured against the risk of exposure to the virus. Often, the patient cohort receiving neuromodulation therapy falls in the vulnerable category, especially the elderly with challenging mobility, comorbid medical conditions, and compromised immune systems^30^. While the COVID-19 pandemic has introduced new challenges, it is worth noting existing gaps in care access that existed previously will remain if such technologies are not available and accessible to patients and clinicians.

This remote care platform is a major milestone to springboard patient-centric neuromodulation innovation. The field recognizes the heterogeneity of patients who are candidates for neuromodulation therapy. Therefore, patient assessments in real-world settings are essential to help clinicians better understand clinical outcomes and aid in appropriate patient selection. Future versions of the remote care platform may be integrated with remote monitoring. Current evidence on using remote monitoring with wearables, on-mobile sensors, and body-worn sensors to classify disease-related states is promising^10,31,32^. An integrated therapy application that is available on the patient’s mobile device is an opportunity to this data as it becomes integral to clinical decision-making in neuromodulation^33^. These data sets can be analyzed to further improve future therapeutics and to identify user-specific gaps and personalize care.

## Conclusion

We summarized the biodesign approach of the design and development of an integrated and wireless remote care platform for a neuromodulation device. Further, we demonstrated the feasibility and characterized the system’s safety in a real-world setting by testing our platform in a clinical study and performing a usability analysis with feature improvements informed by study outcomes. Although the system intended to address pre-COVID19 challenges associated with disease management, the unforeseen overlap of the study with the pandemic has elevated the importance of such a system. It should be considered as an important aspect of the solution for challenges and demands currently facing healthcare. The system functionality and overall experience can be further improved by developing workflows in partnership with experts in the field. Lastly, we are at a pivotal point in the era of human–machine interaction and an insightful ethical discussion is warranted as with any pivotal technological advancement. Future investigations focused on remote platforms should discuss scalability, integration, and access to digital technologies to support patient-centric therapy solutions.

## Data Availability

The authors declare that data relevant to the findings of this study are included in the paper.
